# New insights from combining gait and hand motor function in classification of amnestic MCI and Alzheimer's Disease

**DOI:** 10.1002/alz70856_097359

**Published:** 2025-12-24

**Authors:** Kaylee D Rudd, Katherine Lawler, Michele L Callisaya, James C. Vickers, Jane E. Alty

**Affiliations:** ^1^ Wicking Centre, University of Tasmania, Hobart, TAS, Australia; ^2^ University of Tasmania, Hobart, TAS, Australia; ^3^ La Trobe University, Melbourne, VIC, Australia; ^4^ Wicking Dementia Research and Education Centre, University of Tasmania, Hobart, TAS, Australia; ^5^ Peninsula Clinical School, Central Clinical School, Monash University, Melbourne, VIC, Australia; ^6^ Royal Hobart Hospital, Hobart, TAS, Australia

## Abstract

**Background:**

Gait and key‐tapping are individually associated with mild cognitive impairment (MCI) and dementia. However, it remains unclear whether these motor functions are correlated, or whether combining them improves classification of cognitive diagnoses. We examined 1) associations between motor measures and cognitive diagnosis of Alzheimer's disease (AD), vascular dementia (VaD) and MCI, 2) correlations between gait and key‐tapping measures and 3) whether combining gait and key‐tapping measures improves classification accuracy of cognitive diagnoses over a null model comprising age, sex and education.

**Method:**

Two hundred and forty participants were recruited from a cognitive clinic: 51 with dementia (43 AD, 8 VaD), 106 MCI (68 amnestic‐MCI, 38 nonamnestic‐MCI) and 83 cognitively healthy controls (HC). Consensus diagnosis was made after gold‐standard interdisciplinary assessment, including a neuropsychological battery. Fast‐paced gait was assessed on an electronic walkway and fast‐paced key‐tapping on a computer keyboard. Associations between motor measures and cognitive diagnoses were analysed using Linear regression, adjusted for age, sex and education. Pearson's Correlations were tested. Classification accuracy was calculated using Area under Receiver‐Operating‐Characteristic curves (AUC).

**Results:**

Gait and key‐tapping speed, frequency and variability were associated with AD (*p* <.001) and VaD (*p* ≤ .01). The effect size of the key‐tapping variability was much larger in VaD (β: 225.71) compared to AD (β: 38.30). Gait speed and frequency were associated with both amnestic‐ and nonamnestic‐MCI (*p* ≤ .02). Gait variability (β: .88, P: .008) and key‐tapping variability of either hand (β: 53.01, *p* ≤.02) were associated with nonamnestic‐MCI. Gait and key‐tapping variables were weak‐to‐moderately correlated. Combining gait and key‐tapping speed improved classification of AD (AUC: .95) from HC compared to gait (AUC: .89) or a null model comprising age, sex and education (AUC: .84). The combined gait/key‐tapping speed model also improved discrimination of amnestic‐MCI (AUC: .93) from HC compared to gait (AUC: .81) or the null model (AUC: .74). Combined models did not aid classification of nonamnestic‐MCI.

**Conclusion:**

Overall, slower speed (gait or key‐tapping) was associated with amnestic‐MCI and AD and greater variability with nonamnestic‐MCI and VaD. Weak‐to‐moderate correlations, and improved classification accuracy of the combined gait/key‐tapping model, suggest involvement of distinct motor‐cognitive mechanisms worth further examination.

